# ‘I feel that injustice is being done to me’: a qualitative study of women’s viewpoints on the (lack of) reimbursement for social egg freezing

**DOI:** 10.1186/s12910-022-00774-z

**Published:** 2022-03-29

**Authors:** Michiel De Proost, Gily Coene, Julie Nekkebroeck, Veerle Provoost

**Affiliations:** 1 RHEA (Research Centre Gender, Diversity and Intersectionality), Vrije Universiteit Brussel, Brussels, Belgium; 2 Centre for Reproductive Medicine and Centre for Medical Genetics, UZ Brussel, Brussels, Belgium; 3 Bioethics Institute Ghent, Ghent University, Ghent, Belgium

**Keywords:** Social egg freezing, Oocyte cryopreservation, Assisted reproductive technology, Reimbursement, Qualitative research, Empirical ethics

## Abstract

**Background:**

During the last decade, the possibility for women to cryopreserve oocytes in anticipation of age-related fertility loss, also referred to as social egg freezing, has become an established practice at fertility clinics around the globe. In Europe, there is extensive variation in the costs for this procedure, with the common denominator that there are almost no funding arrangements or reimbursement policies. This is the first qualitative study that specifically explores viewpoints on the (lack of) reimbursement for women who had considered to uptake at least one social egg freezing cycle in Belgium.

**Methods:**

To understand the moral considerations of these women, drawing from twenty-one interviews, this paper integrates elements of a symbiotic empirical ethics approach and thematic analysis.

**Results:**

We identify four themes: (1) being confronted with unclear information; (2) financial costs as ongoing concern; (3) necessity of coverage; (4) extent of reimbursement. In the first theme, we found that some women were concerned about the lack of clear information about the cost of social egg freezing. In the second theme, we report moral sentiments of injustice and discrimination which some women attributed to their struggles and needs not being recognised. The third theme illustrates diverse views on reimbursement, ranging from viewing social egg freezing as an elective treatment not appropriate for reimbursement to preferences for greater public responsibility and wider access. Finally, we describe the participants’ varying proposals for partial reimbursement and the idea that it should not be made available for free.

**Conclusions:**

This research adds important empirical insights to the bioethics debate on social egg freezing, in particular by presenting (potential) users’ views on the lack of reimbursement. While there is much more to say about the ethical and political complexities of the reimbursement of this procedure, our study highlighted the voices of (potential) users and showed that at least some of them would welcome the coverage of SEF through the public healthcare insurance.

**Supplementary Information:**

The online version contains supplementary material available at 10.1186/s12910-022-00774-z.

## Background

During the last decade, the possibility for women to cryopreserve oocytes in anticipation of age-related fertility loss, also referred to as ‘social’ egg freezing (SEF),^1^ has become an established practice at fertility clinics around the globe [1]. The application still raises controversy among bioethicists, policy makers, and other stakeholders. There are ethical concerns about numerous aspects, such as women’s reproductive autonomy [2], medical and emotional risks [3], and the procedure’s individualist and morally problematic dimensions as a solution to the social problems that women face [4].

With regard to SEF, Europe shows a patchwork of policies on this matter and there is extensive variation in the costs for the procedure from one country to another [5]. For instance, in the UK, the cost of SEF is around 3,350 pounds not including medication [1]. In Belgium, the all-in cost (i.e., medication, egg collection, freezing, and egg storage costs) is slightly lower, ranging between 1,500 and 3,200 euros for one cycle.^2^ The common denominator is that there are almost no funding arrangements or reimbursement policies for SEF. In contrast, the cost of egg freezing for cancer patients is reimbursed in several countries, through either direct state funding or a compulsory insurance system such as in the Netherlands.

In 2014, Facebook announced it would cover SEF for its female employees; other *Fortune* 500 companies soon followed. This practice of company-sponsored egg freezing is most common in the US; however, it can be observed in other countries, such as the UK and Belgium [6, 7], if only in limited numbers. Furthermore, there is one small Israeli health fund that partly subsidises the process and in Japan, the city of Urayasu has experimented with a 3-year coverage programme [8, 9].

Dondorp and de Wert expected that SEF could lead to a new round in the debate on coverage for in vitro fertilisation (IVF), challenging the role of the concept of ‘medical necessity’ as a criterion for coverage [10]. Nonetheless, several authors predicted that reimbursement of SEF should not be expected in the near future [11, 12]. This hypothesis was recently echoed in the report of ESHRE on fertility preservation and the Nuffield Council on Bioethics [7, 13]. However, the recent revision of the French bioethics law has defied all predictions and decided to offer partial reimbursement of the clinical procedure costs of ‘non-medical’ egg freezing [14]. Johnston et al. argued that the growing demand for SEF triggers the need for reviewing public funding [15]. There are public arguments for wider subsidies in the popular media of several countries including the UK and Belgium [16, 17]. Recent scientific literature provides further evidence that some medical experts and laypersons are open to the idea of reimbursement [18–22]. While current users seem to be in favour of reimbursement, believing it would enable more equal access [21, 23, 24], some public and professional discourses have been portrayed these women as selfish and hardly concerned about the costs because of their affluent positions and financial security [25, 26].

In the general population, several empirical studies have indicated that the most important barrier for those who would undergo SEF is the prohibitive cost [27–29]. However, inequality of access to this reproductive technology is not limited to costs. Broader, established social hierarchies such as racial identity and sexual orientation may play a vital role in access to SEF [30]. Therefore, the question remains open as to who is likely to benefit from the implementation of reimbursement. As Pennings argues, it could ‘lead to a larger uptake in an already privileged group and might actually further increase injustice rather than diminish it’ [31].

So far, ‘Elective oocyte cryopreservation: who should pay?’ by Mertes and Pennings in 2012 is the only normative study that has analysed reasons for full coverage and other modalities [32]. The authors argued that in a system where IVF is reimbursed, it would be inconsistent to cover IVF treatment with donor eggs for women who are infertile due to ageing while not using their own previously banked eggs for the treatment. Despite the limited uptake of frozen eggs [6], based on concerns about distributive justice there are reasons to argue for full coverage, a cash back-system or greater numbers of free transfer cycles.

Little is known about the views on reimbursement held among women who are currently using the procedure. We started from the premise that including users’ experiences and moral considerations can better contextualise bioethics research and existing normative arguments. As emphasised through the more recent ‘empirical turn’ in bioethics, such an empirical understanding can inform normative argumentation in multiple ways [33]. By drawing on a small qualitative interview study in Belgium, investigating women’s viewpoints on SEF, this study aims to point out previously unrecognised ethical issues, such as single women feeling discriminated against and making access available to women in less privileged financial situations, that have escaped the attention of bioethicists and policymakers thus far. This paper adds a bottom-up perspective to the debate on SEF and enlightens tensions between the current funding scheme operating in Belgium and some women’s viewpoints.

## The Belgian context

Through its mandatory public health insurance, the Belgian government seeks to ensure universal access to basic health care [34]. Although medication and health care services are not for free, citizens and residents can benefit from a basic coverage of their medical expenses if they are a member of an insuring organisation. Doctors can also decide the fees they charge, unless they are known as ‘fund doctors’. In both cases the refund by the healthcare insurance is identical. Supplementary health insurance, the so-called hospitalisation insurance, gives access to a more comprehensive spectrum of covered medical services, especially for hospitalisation claims which can be very expensive due to a large degree of freedom in price setting for medical providers [35]. Generally speaking, persons need to pay for the medical treatment first, and then submit these to the health insurance to reclaim costs. This is not the case for hospital treatment where the hospital charges the health insurance directly and patients pay only their personal share at the end of their hospital visit. Decisions on health care coverage are taken by the Minister of Social affairs following the advice of a multistakeholder appraisal committee, consisting of scientists, healthcare funds, pharmaceutical industry, medical professionals, health care institution representatives, and politicians. Actual users or patients’ associations are not involved in this process [36].

The Royal Decree of 6 October 2008 established that a fixed sum of the IVF costs of up to 6 cycles of treatment are covered under public health insurance [37]. However, coverage only applies if the woman is not more than 43 years old. Unlike other countries, the Belgian law has not established any criterion regarding the profile of the individuals seeking access to IVF. Since 2017 cancer patients, patients with borderline ovarian tumours, and patients with hematopoietic disorders requiring stem cell transplantation, can access public funding for egg freezing and are partially covered [38]. Egg freezing for non-medical or social reasons is allowed yet not covered by the public health insurance. A challenge for SEF is to obtain a relatively accurate estimation of the costs. This is difficult for the reason that many clinics do not mention accurate pricing information on their websites.

## Methods

### Design

For this study, we followed the ‘symbiotic empirical ethics’ approach of Frith, as a meta-ethical background position to integrate social-scientific and ethical analyses [33, 39]. According to Frith, empirical data informs normative theory and vice versa as both mutually adjust each other in a delicate interplay. We implemented Frith’s idea of starting with a more nuanced description of respondents’ reasoning than is commonly found in normative literature. In the discussion section, we further point out theoretical issues that arise from our data. However, within the scope of this paper, we do not make final normative judgements on the practice under study.

A semi-structured interview guidewas developed based on the ethical aspects identified in our systematic literature review [40]. At the beginning of the interview, (open-ended) questions were asked to invite the participants to speak about SEF in their own words. In the second part of the interview, we used elicitation cards with controversial statements covering bioethical issues related to SEF (i.e., autonomy, gender equality, and justice), to encourage the participants to reflect on ethical concerns.Statements were developed on the basis of arguments from the bioethical literature and a full description of the method can be found elsewhere [24, 41]. Participants were asked to articulate their thoughts and we engaged them in Socratic dialogue to investigate discrepancies between their moral understandings and arguments made in the bioethics literature [42]. This paper presents only the data collected on women’s views about reimbursement for SEF. Data on other moral issues related to the elicitation cards are discussed in separated publications [24]. The full interview guide is included in the Additional file 1. 

### Participants

Seventeen participants were recruited by psychologists working in two centres for reproductive medicine at academic hospitals in Belgium where SEF is practiced. The psychologists introduced the study to their patients and asked permission for the first author to contact them. In addition, four participants were recruited through the means of social networks and chain referral sampling. The first author then contacted the patients to schedule interviews. Written informed consent was obtained from all participants before the interview. We interviewed six participants who had successfully completed treatment, twelve who were undergoing the procedure, and three who were interested but undecided at the time of the interview. Table 1 shows the characteristics of the respondents.

**Table 1:** Participants’ backgrounds

*Age (years)*	29–41
Mean age	35
*Relationship status*	
Single	14
New relationship (within six months)	5
Longer relationship	2
*Educational status*	
Bachelor	1
Master	18
PhD	2
*Nationality (citizenship)*	
Belgian	15
Brazilian	1
Egyptian	1
French	1
Dutch	1
New-Zealand	1
Ugandan	1
*Sexual orientation*	
Bisexual	1
Heterosexual	19
Lesbian	1
*Religion*	
Catholic	2
Christian	5
Muslim	1
None	13
*Net income (euros)*	
750–1500	1
1500–2000	3
2000–3000	12
> 3000	5

### Data collection

In total, we conducted twenty-one interviews. All interviews were performed by the first author and took place between February 2019 and November 2020 at a location of the participants’ preference (*n* = 11) or through online video connections (*n* = 10). The interviews ranged from 40 min to 2 h. They were conducted in three different languages (Dutch, English, and French). Each interview was audio-recorded and transcribed verbatim by the first author using pseudonyms in the transcripts, which were then checked for accuracy by the other authors. Below, we present participants’ (pseudonymous) quotes and, with each quote, the age of the participant and a letter code showing whether the participant had already frozen her eggs (f) or was still weighing options prior to freezing (pf).

### Analysis

The data from these interviews were analysed using a reflexive thematic analysis combined with interdisciplinary collaborative auditing designed for empirical ethics projects [43, 44]. The first author carried out the initial coding and, with the assistance of QSR International’s NVivo 12 data analysis software, organised the codes into a potential thematic map. Coding summary reports were sent to the auditors, listed here as co-authors, in advance of team meetings. Based on these reports the auditors challenged the themes and subthemes constructed by the first author. The collaborative reflection of this auditing process was repeated several times until no further exploration would result in new insights, and it significantly enhanced the validity and rigour of the analysis, resulting in a more reflexive reading of the data.

## Results

We formulated four main themes that interpreted participants’ concerns and moral considerations regarding the reimbursement of SEF. The first two themes were constructed to show their spontaneous reactions and experiences to the lack of state-funded reimbursement. The third theme was developed to show their viewpoints on whether or not governments should cover this particular treatment, and the fourth theme was generated around participants’ various concrete proposals for reimbursement. These four themes are illustrated below.

### Being confronted with unclear information

During the interviews we observed several inconsistencies in the information and understanding participants had regarding the price and the possibility of reimbursement. They were unsure about whether the procedure would be reimbursed through their health insurance. For instance, Kaat (36, pf) said, ‘I heard recently that a health insurance can cover a lot and may reimburse egg freezing’. Or, as Lotte (35, f) reported, ‘there are some [hospitalisation insurances] that will reimburse it, but I have asked the question and they said no’. One participant narrated that in the clinic she had received unclear information about the topic:

A lot of information was given but the information about, how do you call it, whether or not you could get something from your health insurance, was rather ambiguous at [clinic]. They made it seem that you could get some money for the operation. [. . .] I’ve made inquiries about that at my [insurance], but it turned out not to be the case. (Isla, 38, f)

Maud (38, pf) described a similar story of health care professionals who indicated ‘that the costs could be reduced’.

The lack of clear information was troubling for some of the participants: as Isla (38, f) said, ‘I’m not quite sure why they made it seem that I would be able to get something back’. Several participants were startled and even shocked when they first heard the price of the procedure during medical consultations. This was often related to experiences of miscommunication: as Martine (33, pf) told us, ‘my general practitioner said, “I think it’s 1300 euros,” and then they [clinic] said, “it’s 2700 euros”—that’s suddenly twice the initial price’. Julie (34, f) highlighted how in one clinic the informational forms used for infertility treatment had not been updated and tailored to the specific situation of SEF: ‘I got a form that was meant for a couple, and it had a price of 400 euros on it; during the consultation, the doctor crossed that out and wrote, for you that’s 3200 euros.’ It seemed the participants expected more guidance from the clinics and wider dissemination of information. Left to discover the information on their own, some participants expressed feelings of self-doubt in words such as Isla’s (38, f): ‘perhaps I have not been able to investigate it sufficiently’.

### Financial costs as ongoing concern

Some participants compared SEF prices to find the cheapest procedure and appreciated efforts of clinics to reduce costs. Lotte (35, f) revealed that ‘in [clinic] they actually persuaded me by saying “you mustn’t hesitate” and when she said the price, I thought “okay, I think it’s a bit cheaper than in [clinic] so let’s just do it here.”’ According to Maud (38, pf), ‘There was also high financial pressure from [clinic]; it is only at [clinic] that a cheaper formula was discussed.’

Although the reduction of costs emerged as a central theme for some participants, others took a more enterprising way of choosing a hospital. Lan (35, pf) used the metaphor of a housing renovation: ‘If you need advice from someone about your rooftop or other housework, you just ask for several quotations; it’s the same thing.’ Moreover, some expressed the desire to pay more to get better quality: ‘If you want to buy a car and it is an extra 1000 euros to have more options, good quality and one that is more durable, you would pay 1000 euros more’ (Elmira, 38, pf).

Some participants indicated that worrying about costs delayed their decision. The following example illustrates this dynamic:

If it was much cheaper, at 34, I think I would have done it at that age because I have had better quality eggs. The high cost makes you overthink, makes you delay everything—in my case anyway—until you’re reaching a limit (Isla, 38, f).

Isla’s comment alluded to the increasing worry about high costs while passing time may mean lower chances of success with eggs frozen after delaying the decision. Martine (33, pf) described how ‘financially, it is a very big cost’ and she thought about the question: ‘How much is that worth to me?’. In a similar vein, Kato (33, pf) narrated that ‘4000 euros is not a small amount to have it done now and perhaps never need it’.

Other participants illustrated how they tried to manage the monetary costs: Erika (37, pf) said ‘luckily, I have financial independence so I can pay for it and if I couldn’t, I could certainly fall back on my parents who could also help me’. Maud (38, pf) said, ‘I’m going to take it from the money I got from my parents to buy a house, which was already not that much but enough.’ Though almost every participant described the procedure as Lan (35, pf) did—‘super expensive’ –, some participants indicated how the financial cost was not really a worry for them. As Julia (37, f) put it, ‘the money is not going to make any difference in my life’. Martine (33, pf) said, ‘I have a reserve; it is not a question of survival for me; I can still continue to live and go on with everything I do.’

Even though most of the participants in this study indicated they could afford the costs of SEF, several emphasised that they did not go for a second cycle because they found it too expensive. It seemed that, for them, the extra cost outweighed the benefit of having more eggs frozen. Nina (33, f), for instance, said ‘I’ve wondered whether I would have done it a second time if it were less expensive; [...] to me, the additional value is not worth the high cost’. In Laura’s (31, f) words, ‘It would again be something like 3000 euros and I am not willing to pay that in order to add, let’s say, that thirty percent chance’.

Several participants reported how they felt subjected to injustice when considering the lack of reimbursement. ‘My gut feeling is that it’s a bit unfair,’ said Julie (34, f). ‘I just think it’s unfair that it’s not reimbursed, that you’re excluded from something you’re entitled to as a woman,’ said Emma (35, f), going on to say, ‘I feel that injustice is being done to me’. Emma also argued that almost everything is reimbursed in Belgium, ‘at the gynaecologist you get 10 euros back [after a check-up]’, but not for this treatment. For some participants it was, therefore, hard to understand why the government would not reimburse them for this specific procedure. Laura (31, f) criticised this policy as puzzling by saying ‘I actually don’t know why the government doesn’t do it’ while Julie (34, f) said ‘I don’t really understand the logic behind it’. They paralleled their own situation to patients undergoing other fertility treatments, such as regular IVF procedures: ‘you almost get punished,’ said Lotte (35, f). Martine (33, pf) said of regular IVF patients, ‘they were just lucky enough to have a partner,’ and Julie (34, f) questioned this arbitrariness: ‘I don’t see why I should pay much more than couples.’ In these views, single persons opting for egg freezing are denied the opportunity for reimbursing some costs related to the process, and some participants therefore perceived this as a form of discrimination.

Some participants were less disappointed and expressed hesitation about whether they could speak of discrimination in their cases. ‘I would not push it that far because it is so two-sided for me,’ said Erika (37, pf), just as Kato (33, pf) noted that ‘you can say on the one side yes and on the other side no’. Nina (33, f) argued that her singlehood was a consequence of choices while IVF was more of a necessity, yet she positioned herself as a ‘strong empowered woman’ and someone who had ‘luxury problems’. In a similar way, Elmira (38, pf) found she was not treated ‘unfairly’ because the whole procedure was a form of ‘self-investment’.

### Necessity of coverage

Participants tried to conceptualise the elusive parameters of what constituted necessary medical treatment to indicate which interventions should be reimbursed by the health insurance system. The difference between therapeutic and elective treatment could not be neatly drawn. When asked if she would describe her case as a medical necessity, Lotte spoke as follows:

A little bit, social freezing—not if I did it at 25, but now they are saying ‘your stock is running out’. Of course, the doctors can’t do anything about the fact I don’t have children yet; that’s my own choice, so I understand that. No, I am not medically unhealthy—or I have no problems in this regard—so I think it is a very difficult debate. (Lotte, 35, f)

Martine (33, pf) pondered the issue in a similar vein, saying, ‘you can have very few eggs by nature, not being fertile is what I would call a disease, so I was like, what do they define as a disease?’ Some participants, especially if they had previous medical conditions that influenced their current situations, perceived themselves as patients asking for a medical intervention and therefore deserving some form of reimbursement. One interviewee provided the following example:

I had a serious HPV infection, so I had to deal with that for many years. In my case I see that as the reason why I got into this in the first place, and I think it’s actually unfair that as a woman you still have to pay a lot of money. (Isla, 38, f)

Other participants focused on the idea that they did not have medical problems and were completely healthy: ‘I don’t have a medical problem,’ said Nina (33, f); ‘I have a luxury problem. I’m medically healthy, I just have a mismatch at the social level for the moment, but I don’t have a medical problem.’ These participants did not underestimate the psychological impact of being single and childless. However, in their view, this fact did not itself provide a justification for reimbursing.

Some participants drew on popular understandings of cosmetic surgery to legitimate their views on reimbursement for SEF. Two participants saw clear similarities with their own procedure, Melissa (41, f) referring to facelifts and Nina (33, f) saying she thought users of cosmetic surgery had similar intentions to hers in wanting to ‘buy a feeling’. In her case, the search for peace of mind overlapped with the self-confidence that someone would seek if they did not feel right about their nose. This sparked the insight that she would see no good reason for reimbursing SEF.

Other participants demonstrated confusion because of what they saw as vague distinctions between SEF and plastic surgery. It seemed comparable but was also significantly different, in a way, from their own treatment. One participant expressed this confusion as follows:

It’s a personal choice; it’s like plastic surgery—the government is not going to intervene in that either, nor is health insurance. That’s also a choice you make for yourself. But I think this is like a little bit more based on medical necessity. (Lotte, 35, f)

Maaike (35, pf) held the view that ‘a beauty problem can be a psychological burden for someone—that can weigh heavily on you—and can be medically treated’. She said her experience with egg freezing included a similar psychological experience although egg freezing ‘is about a child; it’s not about if I look pretty today’. Because egg freezing was related to ‘your womanhood, to your biological metabolism’, for Laura (31, f) it was clearly distanced from cosmetic surgery’s more aesthetic focus.

During the interviews, participants reflected on how the government must set priorities for reimbursing in times of shortage of funds. A few seemed to indicate they were not entitled to ask for public money: Melissa (41, f) said ‘I have a lot in my life; so you don’t have a child. Other people don’t have other things.’ Since resources are limited, she said, the state is not obliged to support each particular life project and related needs. Melissa continued:

The context is always scarcity; if you have to choose between reimbursement for this [egg freezing], reimbursement for a cancer treatment or plastic surgery [. . .] then it gets interesting because you might imagine we have a cake. We only have a limited number of slices. Who should receive a bigger slice of the cake? Ideally, everyone deserves to be happy and should have access to this. (Melissa, 41, f)

Melissa seemed to hold the view that the morally right action is the one that maximises happiness; she indicated that we need to determine whose needs are more ‘deserving’ of those limited resources. In a similar vein, Jie (33, pf) said ‘I don’t know what the government would try to solve in this case, [...] but if their act creates harm to society as a whole and reduces the total good, then it is unethical.’

Some participants found that the needs of women were not taken into account when the question of priority was asked. Moreover, the argument of scarce resources was perceived relative to circumstances: ‘I think there are many other things in society that are costly too and where you can get money from’ (Laura, 31, f). In her view, the added costs of reimbursements for SEF might not be overwhelming and could be funded instead of other societal expenditures.

Because societal trends that are beyond individual control, such as the rise of highly educated professional women, were at the basis of the postponement of motherhood, it was evident for some participants that society should take responsibility and support this group of women. For example, Laura remarked as follows:

If you want more people to get an education [. . .] you also graduate and start working later; having a child is also delayed by this chain of events. So, it seems logical to me that in the light of these things they encourage, this is an option as well. (Laura, 31, f)

Jie (33, pf) indicated ‘globally in the last couple of years, the number of egg freezing cycles has increased a lot so there is a need for it and I think based on this natural part that governments should take care of it’. Laura (31, f) further declared: ‘You are actually disadvantaged as a woman and as long as that disadvantage is there, I think society should also do something in return.’

Several participants found that no public funding was necessary because of possible manipulation and the circumvention of boundaries between the ‘natural’ reproductive lifespan. For instance, Maud (38, pf) said ‘I still think there are limits to the malleability of human beings and that we should avoid having miles of freezers with eggs that are never used.’ And Kato (33, pf): ‘Sometimes I wonder about the future, infertility that is going to be even more and more, I think [...] stop inventing things because it all goes too far.’ These thoughts about egg freezing were preoccupied by doomsday scenarios about a future where only ‘unnatural’ reproduction would exist. Other participants were rather sceptical about these claims and made the following counterargument: ‘women are not just going to take this step. [...] It’s serious stuff you’re thinking about,’ said Isla (38, f), ‘It’s going to stagnate at some point’, said Laura (31, f). Furthermore, as Lan (35, pf) described it, ‘I think it is not an option to recruit everyone who doesn’t have kids to have frozen eggs anyway. It’s more like, if you are in the maybe group, it should be possible for you to do it.’

Kaat used a similar argument, that social egg freezers would make up only a very small minority:

I think most people have children much earlier, so it’s a small target group anyway. So, you’re not going to stimulate more people to do this. I think there are just as many people who are 38 and don’t want to have kids. (Kaat, 36, pf)

Kaat added that the financial aspect is not the only thing that matters to people. In her view, if you really have a desire for children, you are often willing to spend a lot of money for this unless you have no money at all. In this regard, some participants were troubled by how the costs of SEF could limit access for women with other profiles like ‘working-class’ as Nina (33, f) put it or, in Melissa’s (41, f) words, ‘women of colour’. They imagined how others’ well-being was affected by this procedure. As Erika (37, pf) described her thinking, ‘I immediately made the reflection, I wonder if all women could pay for this’; or, as Julia (34, f) put it: ‘Why should privileged people have the opportunity and others not?’ Therefore, several participants assumed that governments should reimburse the procedure simply for the reason of stimulating broader access.

### Extent of reimbursement

Most participants suggested covering only a part of the procedure through reimbursement: Lan (35, pf) suggested ‘maybe half; for example make it cheaper, really cheaper. I am not asking that they reimburse 2500—3000 euros’. ‘I wouldn’t say a full reimbursement but maybe a large portion,’ said Annemie (36, f), while Lotte (35, f) said ‘I understand that they don’t intervene completely as with IVF, but a partial coverage might be possible’.

Some participants were in favour of a system that would differentiate based on wage level when it comes to reimbursement. According to Maaike (35, pf), only people or families whose income is below a particular threshold should be reimbursed for egg freezing. She said, ‘I think you can take it into account in the healthcare system; for example, someone who is less well-off might be eligible for a reimbursement’. As another interviewee supporting this view, Laura indicated the following:

You could also tailor the reimbursement system to the level of income. It may be that in fact you earn too much, and you don’t get reimbursed. So, you could work with a percentage, to provide opportunities for people who might just not think about it because it’s so expensive. (Laura, 31, f)

For some participants the idea of partial coverage seemed morally sound, but they were opposed offering this procedure for free. The following quote shows Nina’s position:

Not for free, I’m against this idea. [. . .] If something is free, it has a different value for the recipient. If I give you a free book or you had to give 50 euros for the same book, you’re going to value the book more at 50 euros. So, to keep us aware and respectful, it should not be free. But 2500 [euros] per cycle is too expensive. You exclude too many women. (Nina, 33, f)

Providing SEF as a free good, in these participants’ experiences, would act as a catalyst for the devaluation of the procedure: ‘people may only do this because it is free’ (Lan, 35, pf). Having children was thought to be a unique good that required certain preconditions and effort, as Annemie argued; it was not interchangeable with something that you got for free. For these participants, broader access to egg freezing was necessary but it should not be made effortless.

Unlike the others, two participants were in favour of leaving things as they were (women paying fully out-of-pocket for the procedure) and did not find it legitimate to reimburse the procedure. Melissa (41, f) indicated she ‘would prefer if this were expensive, rather than a cancer treatment’. Moreover, Jie (33, pf) confessed, ‘Honestly I don’t know if there is a problem because women who consider doing this, I suspect, have an income level that is more than enough to cover the cost.’ In her view, the lack of reimbursement was acceptable because current users can financially self-support their efforts.

## Discussion

A primary issue in the SEF debate is whether the community ought to devote scarce healthcare resources to it [2]. Our study adds to this debate by identifying several distinct viewpoints on this topic. This study sheds light on how the current lack of reimbursement was perceived among women who wanted to initiate or had undergone at least one egg freezing cycle in Belgium. While women’s concerns regarding the costs of freezing have often been mentioned as a side note in empirical studies on women’s motivations to freeze their eggs [21, 45], our study is one of the very few studies who investigated the topic in a systematic manner [15].

The study showed that some participants struggled to access the level of information they deemed necessary in order to make an informed and autonomous choice, in particular information about the cost of the procedure [2, 3]. This lack of clarity regarding the true cost and reimbursement options may impact the possibility for women to budget appropriately regarding what is already an expensive procedure. Moreover, our findings showed how participants gathered information through sources other than health care providers, such as health insurance funds, in order to establish further understanding of the reimbursement options. This process added decision stress for some participants because of the administrative burden, a burden that could easily be avoided if clear information could be provided—for example, on clinic websites or during consultations. Other studies have also indicated it is particularly worrying that pricing information is usually unclear [46, 47]. Greater transparency and in particular standardised presentation of reimbursement options could remedy that.

Our results further illustrate how participants seem to differ in the weight they attach to the cost as a factor in their decision whether or not to freeze their eggs. For some women the monetary costs appeared manageable, not really a concern; other women were troubled by the price tag and were prone to the advertisements of service providers who offered cheaper or better services. They coped with the cost in several ways, mainly by delaying their decision or reducing the number of freezing cycles. This prompts the question: How do women weigh the personal benefits of SEF in relation to the money they would need to spend? It seems not all participants wanted to go to the same lengths and complete the clinically advised number of cycles for maximum success with frozen eggs to achieve a future pregnancy. This finding is contrary to previous empirical studies, which suggested women make such decisions irrespective of the costs [21, 45].

In accordance with previous qualitative studies on SEF [23, 48], we found that some participants felt discriminated against while reimbursement was not available. It is striking that some, being single, felt discriminated against based on their relationship status whereas Belgian compulsory health insurance covers single women in the same way as women in relationships if they undergo a full IVF procedure [49]. Participants were baffled by this policy and contested the given distinction. That is, they found it inconsistent that the state offers almost unlimited reimbursement for IVF but remains insensitive to their needs or preferences.^3^

In practical terms, SEF patients differ from IVF patients in that they require a twofold procedure. First, they need to have their eggs frozen because they do not feel ready and eager to pursue parenthood; second, they may or may not return (often years later) to have their eggs thawed and fertilised and then transferred. This leads to feelings of frustration and misrecognition. In addition, many studies around the world have found that women’s decision to postpone motherhood is not exclusively a conscious decision but is based on variables beyond their control such as finding a committed partner [1, 50–53]. This observation could support the hypothesis that these women are unfairly disadvantaged in the allocation of public resources. It is interesting that we found these feelings especially among the women with the lowest net income (below 3000 euros) in the total group of interviewees. However, our sample is too small to make a valid statement about a possible correlation. Future research needs to explore the possible relationship between socioeconomic status and views on reimbursement.

Our results suggest different views on whether SEF could be considered a medical need or not. Some participants were reluctant to frame their decision as a question about a medical intervention, as they felt SEF was based on personal preference rather than related to a medical condition. They were sceptical about reimbursement for a treatment that, like cosmetic surgery, was intended to address social needs or psychological discomfort rather than physiological conditions. Others saw SEF as a response to a medical problem or, at least, as a treatment based on medical advice. These findings confirm previous work that has pointed to considerable ambiguity in recognising a (medical) need for this intervention [15, 54, 55].

In this study, the tension between potential users’ perception of personal responsibility for timely family planning and their need or preference for increased public coverage of SEF became apparent. Some participants, who seemed to show traces of neoliberal discourse in their reasoning [50, 56], emphasised personal responsibility for their SEF decisions and felt that this procedure should be considered a private matter that does not require public intervention. This reflects similar findings in a recent study by Kaplan, Hashiloni-Dolev, and Kroløkke, who found that most female students in Israel and Denmark supported self-financing of SEF [22].

In our small sample, however, the scale seemed to tip towards a preference for reimbursement, as most of our participants suggested public financing of SEF. One reason for this preference was the underlying perception that SEF was a consequence of deeper societal issues. These include the question of growing numbers of higher-educated professional women as well as demanding work conditions that preclude an acceptable work-family balance, and the difficulties higher-educated women face in finding suitable partners. These women thus indicated, as a result of the societal problems they needed to tackle, they would make a claim asking society for financial support of their individual efforts to anticipate age-related fertility loss. Another reason these women were in favour of reimbursement was that this would increase the affordability of SEF for other women. Their moral stance on the issue of access to SEF appeared to mark a sense of solidarity with women whose access to the treatment is limited. It has been suggested that women opting for SEF are primarily focused on the realisation of self-centred preferences [25, 57], but when they discuss the topic of reimbursement, they at least seem to be concerned about more than merely their own needs.

The main strength of our study lies in the in-depth examination of the women’s views and their moral reasoning on the topic of reimbursement for SEF. Notwithstanding, some limitations need to be discussed. Firstly, our study is limited in that it reflects viewpoints of women who succeeded in accessing treatment. Women who decided against SEF (possibly due to its cost) were not included in the study. Future research should try to reach out to a more diverse sample and to include the perspectives of other stakeholders, including policy makers and health care professionals, on reimbursement. Secondly, some women may have been uncomfortable with being interviewed on their choices about a highly personal and intimate matter. This may have resulted in offering what they deemed more socially desirable answers. However, we carefully analysed how participants responded to the interview questions, especially to the Socratic-style questions. With this in mind, we want to stress that our results are a form of co-constructed knowledge. Finally, the specific type of data collection used for this study can never fully capture people’s viewpoints on reimbursement. Other data sources, such as policy documents, newspapers, and social media discussions will be important for achieving a fuller understanding of the controversy under study.

## Conclusion

The debate on reimbursement of SEF has been going on since Mertes and Pennings raised the question of who should pay for SEF almost ten years ago [32]. However, there is still a need to hear women’s voices on the topic as they are not consulted to inform health care coverage decisions. This research yields important empirical insights that is rooted in women’s real-world views and should inform further discussion on whether or not some form of reimbursement is morally acceptable. We found that some women were concerned about lack of clear information on the cost of SEF. They also reported moral sentiments of injustice and discrimination which they attributed to the lack of recognition for their struggles and needs. Other women perceived the controversy surrounding the reimbursement for SEF as something far removed from their lived experience. Based on our findings, it seems to be an oversimplification in some discourses to portray women interested in SEF as merely affluent and unconcerned about the coverage of egg freezing costs.

While there would be much more to say about the ethical and political complexities of reimbursement for this procedure, our study has highlighted the voices of (potential) users and shown that at least some of them would welcome the coverage of SEF through the public health insurance system. To be clear, we are not suggesting that reimbursement policies should follow the views we found. What we want to point out with this study is that the ongoing debate and further research should pay attention to the voices of women who have direct experience of SEF.

## Supplementary Information

**Additional file 1**. Semi-structured interview guide.

## Data Availability

The dataset used and/or analysed during the current study is available from the corresponding author on reasonable request.

## Abbreviations

ESHRE: European Society of Human Reproduction and Embryology
IVF: In vitro fertilisation
SEF: Social egg freezing

## Footnotes

1 Different conceptualisations of social egg freezing, such as ‘elective egg freezing’ or ‘anticipated gamete exhaustion (AGE) banking’ [23, 55], were constructed to highlight the practice’s normative connotations. However, in this paper we use the term ‘social egg freezing’ because it is commonly used in European discourse.

2 This information is based on the participants we interviewed for this study.

3 Whether SEF is a need or merely a preference is an interesting question, but a full discussion of this philosophical issue lies beyond the scope of this study’s empirical perspective. The primary focus has not been a normative perspective but how women actually reason. For work that takes this perspective, see for instance Segers et al. [58]. We do not draw a strict distinction between these two terms because this reflects how the terms were used by the participants.
