# Supplementary material for: ‘I feel that injustice is being done to me’: a qualitative study of women’s viewpoints on the (lack of) reimbursement for social egg freezing
Source: BMC Med Ethics. 2022 Mar 29;23:35. doi: 10.1186/s12910-022-00774-z (PMC8966350; doi:10.1186/s12910-022-00774-z)
Supplement: Supplementary file 1 — Additional file 1. Semi-structured interview guide. [file 12910_2022_774_MOESM1_ESM.docx]

**Additional file 1. Semi-structured interview guide**

Part 1: personal experience with SEF

1. Motivation, reasons for freezing
   1. When did you began to think about egg freezing and why?
   2. How did you find out the existence of this technology?
   3. What was it about egg freezing which appealed to you? Did you consider any alternatives to egg freezing?
   4. What was it about your situation which made you feel that becoming a mother was not yet something you felt able to pursue?
      1. What does parenthood mean to you? What do you think about single motherhood?
      2. Which conditions are important to you? Income? Relationship? Stability?
   5. Which factors had an influence on your choice to start with this procedure?
2. Relational aspects
   1. Did you discuss this with anyone?
   2. Have their opinions influenced your decision?
   3. Was there anyone you didn’t tell?
   4. Do you know anyone else who has frozen their eggs or may have thought about it?
3. Experience with the clinic and process
   1. How did you choose a clinic?
   2. What was your experience of that clinic like?
   3. What kind of information did the clinic discuss with you before starting the procedure?
      1. Do you find the given information sufficient?
      2. Have you also looked for information on your own?
      3. What information would you give to other candidates?
   4. Can you tell me about your experiences with the process at this moment?
      1. What did you find the most difficult and what appears to be better than expected?
      2. Did you ever consider stopping? What made you continue?
4. Self-perception
   1. Has freezing your eggs made a difference to how you see yourself?
      1. Prompts: If so, how do you see yourself now as different from before you started with egg freezing? How would you say you have changed?
   2. What about the way other people see you?
      1. Prompts: members of your family, friends? Changed?

Part 2: Statement cards

| Statements | Description of relevant moral issues in the SEF debate |
| --- | --- |
| ‘Every woman should have access to this technology.’ | ‘access’ statement probing the question on who should have access and whether this is the current reality |
| ‘Egg freezing is an individual and technological solution to a social problem.’ | ‘individualisation’ statement probing the question that it is morally problematic for individuals to use technology to handle a problem that is social in nature |
| ‘Egg freezing leads to the further oppression of disadvantaged groups in society.’ | ‘further oppression’ statement probing the question if this technology worsens the oppression of disadvantaged groups (women, people of colour, sexual minorities) |
| ‘Egg freezing gives women more freedom.’ | ‘freedom’ statement probing questions on the emancipatory potential of this technology |
